# Herpesvirus lytic infection-induced mitophagy via viral interferon regulatory factor 1

**DOI:** 10.1080/27694127.2023.2281135

**Published:** 2023-11-14

**Authors:** Mai Tram Vo, Young Bong Choi

**Affiliations:** Department of Oncology, Sidney Kimmel Comprehensive Cancer Center, Johns Hopkins University School of Medicine, Baltimore, MD 21287, USA

**Keywords:** BNIP3L, Human herpesvirus 8, GABARAPL1, Mitochondria, Mitophagy, Nix, TUFM, vIRF-1, Kaposi sarcoma-associated herpesvirus

## Abstract

Viral control of mitochondria via mitophagy has a dampening effect on mitochondrion-mediated innate immune responses. We previously found that human herpesvirus 8 (HHV-8) could activate mitophagy via its lytic gene product vIRF-1 (viral interferon regulatory factor 1). Mechanistically, we previously demonstrated that vIRF-1 interacts with the mitophagic proteins BNIP3L (BCL2 interacting protein 3 like) and TUFM (Tu translation elongation factor, mitochondrial). Despite these significant findings, however, the precise molecular mechanisms underlying vIRF-1-activated mitophagy, particularly with core components of the autophagy machinery, remained to be fully elucidated. We recently reported that vIRF-1 binds preferentially and directly to GABARAPL1 (GABA type A receptor associated protein like 1) in a noncanonical manner, and this interaction is essential for virus-productive replication. Furthermore, we found that BNIP3L is a crucial factor that promotes vIRF-1 oligomerization and associated mitophagy activation, including GABARAPL1 interaction with vIRF-1 and TUFM dimerization. Together, our findings deepen our understanding of lytic infection-induced mitophagy and provide the key protein-protein interactions involved in vIRF-1-mediated mitophagy.

**Abbreviations:** ATG8, autophagy-related gene 8; BNIP3L, BCL2 interacting protein 3 like; HHV-8, human herpesvirus 8; GABARAP, GABA type A receptor associated protein; GABARAPL1, GABARAP-like 1; LC3, microtubule-associated protein 1 light chain 3; LIR, LC3-interacting region; Nix, NIP3-like protein X; TUFM, Tu translation elongation factor, mitochondria; vIRF-1, viral interferon regulatory factor 1; UIM, ubiquitin-interacting motif; VIR, vIRF-1-interacting region.

In addition to their role in energy metabolism, mitochondria act as an antiviral signaling hub that mediates innate immune responses, including apoptosis and type I interferon expression, in response to pathogen infections. Moreover, the accumulation of damaged or dysfunctional mitochondria increases oxidative stress, which can potentiate apoptosis through mitochondrial membrane depolarization and augment type I interferon signaling. However, viruses have evolved strategies to attenuate mitochondria-mediated antiviral signaling for their successful infection and replication. Recent studies have demonstrated that viruses can eliminate infection-altered mitochondria via the selective autophagy of mitochondria, termed mitophagy, leading to an attenuation of the antiviral innate immune responses.

Human herpesvirus 8 (HHV-8), also known as Kaposi sarcoma-associated herpesvirus (KSHV), is the etiological agent that is causally associated with human malignancies, including Kaposi sarcoma, primary effusion lymphoma, and multicentric Castleman’s disease, in immunodeficient individuals. Like other herpesviruses, HHV-8 has two distinct infection stages in the host: latency (persistent infection) and lytic replication, both of which are related to HHV-8 pathogenesis. We previously demonstrated that mitophagy is activated upon lytic reactivation via HHV-8-encoded vIRF-1 (vial interferon regulatory factor 1), which is expressed highly during lytic replication and localizes in part to the outer mitochondrial membrane via its N-terminal region. We also found that vIRF-1 interacts with the mitophagic proteins BNIP3L (BCL2-interacting protein 3 like), also known as Nix (NIP3-like protein X), and TUFM (Tu translation elongation factor, mitochondria) and, thereby, inhibiting mitochondria-mediated apoptosis elicited by lytic reactivation, which in turn promotes lytic productive replication of HHV-8.

To better understand the molecular mechanisms of vIRF-1-mediated mitophagy, we examined whether vIRF-1 interacts with the ATG8 (autophagy-related gene 8) protein family proteins, MAP1LC3A/LC3A (microtubule associated protein 1 light chain 3 alpha), LC3B, LC3C, GABARAP (GABA type A receptor associated protein), GABARAPL1 (GABARAP-like 1), and GABARAPL2, because vIRF-1 is predicted to contain three regions with the consensus sequences of the LC3-interacting region (LIR) motif by *in silico* inspection with the web-based tool iLIR. *In vitro* binding assays using purified proteins showed that vIRF-1 binds preferentially to LC3C, GABARAP, and GABARAPL1^1^. Moreover, co-immunoprecipitation and immunofluorescence assays showed that vIRF-1 could associate with only GABARAPL1 and, to a lesser extent, GABARAP, at the mitochondria of HHV-8-infected cells. However, the predicted LIR motifs of vIRF-1 were not involved in the interaction with GABARAPL1, suggesting an LIR-independent binding of vIRF-1 to GABARAPL1. Therefore, we sought to map the binding sites involved in the vIRF-1 and GABARAPL1 interaction using *in vitro* binding assays. We could identify the region encompassing amino acids 227 to 236 of vIRF-1 as the GABARAPL1-interacting region. These sequences are different from the ATG8-interacting UIM (ubiquitin-interacting motif)-like sequences. Also, we found that the residues of glutamic acid at position 17, lysine at 20, and lysine at 38 of GABARAPL1 are required for the interaction with vIRF-1. The vIRF-1-interacing region is located out of the LIR and UIM docking sites of GABARAPL1. Thus, the vIRF-1:GABARAPL1 interaction represents a novel class of ATG8 interaction.

To examine the functional relevance of the interaction between vIRF-1 and GABARAPL1 in HHV-8 lytic replication, the GABARAPL1-interacting region (227-236) of vIRF-1 was deleted in HHV-8 bacterial artificial chromosome 16 (BAC16), and iSLK cells were infected with BAC16 and the mutant (BAC16.vIRF-1Δ227-236), This approach showed that the levels of the mitochondrial protein MT-ND1 decreased in iSLK.BAC16 cells, but not iSLK.BAC16.vIRF-1Δ227-236, following reactivation of HHV-8. Moreover, the production of encapsidated HHV-8 virions significantly decreased in reactivated iSLK.BAC16.vIRF-1Δ227-236 cells compared to reactivated iSLK.BAC16 cells. Together, our results suggest that vIRF-1 interaction with GABARAPL1 plays a critical role in the mitophagic regulation of mitochondria levels and HHV-8 replication.

We previously demonstrated that BNIP3L is critical for vIRF-1-activated mitophagy. Thus, we sought to examine whether BNIP3L, which is a selective mitophagy receptor that binds to ATG8 proteins via its LIR motif, has an effect on the vIRF-1-GABARAPL1 interaction. Indeed, co-immunoprecipitation and NanoBiT protein complementation experiments revealed that BNIP3L expression promoted the interaction between GABARAPL1 and vIRF-1. In addition, mitophagy flux assays using the mitophagy reporter ‘mito-mCE’, in which the mCherry-EGFP tandem fluorescence proteins are fused to TOMM20 (translocase of outer mitochondrial membrane 20) N-terminal residues 1-33 for mitochondrial targeting, showed that co-transfection of GABARAPL1 together with both vIRF-1 and BNIP3L led to a much higher ratio of red to green fluorescence compared to single or double transfection with vIRF-1, BNIP3L, and GABARAPL1. Consistent with this, vIRF-1/BNIP3L-mediated mitophagy was significantly reduced in GABARAPL1-deficient cells. Together, these results strongly suggest that the three proteins form a complex promoting HHV-8 reactivation-induced mitophagy.

In addition, we found that overexpression of BNIP3L could induce mitochondria localization and aggregation of vIRF-1 in transfected cells, and the levels of the aggregated vIRF-1 were greatly reduced in BNIP3L-deficient BCBL-1 (HHV-8-infected) cells with HHV-8 infection at levels similar to those of control cells after reactivation. Importantly, the levels of autophagy-competent TUFM, a dimerized form induced by vIRF-1 expression, were reduced in the BNIP3L-deficient BCBL-1 cells. These results suggest that BNIP3L is upstream of vIRF-1-mediated, TUFM-mediated mitophagy in lytically HHV-8-infected cells.

GABARAPL1 is known to interact with proteins in the multiple steps of autophagy, including autophagosome initiation, closure, and fusion with lysosomes (Figure 1). Thus, it is conceivable that the BNIP3L/vIRF-1 complex may be involved in the entire autophagy process via GABARAPL1, from autophagosome biogenesis to fusion with lysosomes, especially for mitophagy during lytic replication (Figure 1). Further studies are warranted to examine whether vIRF-1 is indeed involved in all the different steps of autophagy via GABARAPL1. Findings from our recent study could be highly significant because they may provide a model in which the virus can activate proviral mitophagy by recruiting the core autophagy machinery to particular cargo, such as mitochondria.

**Figure 1.**
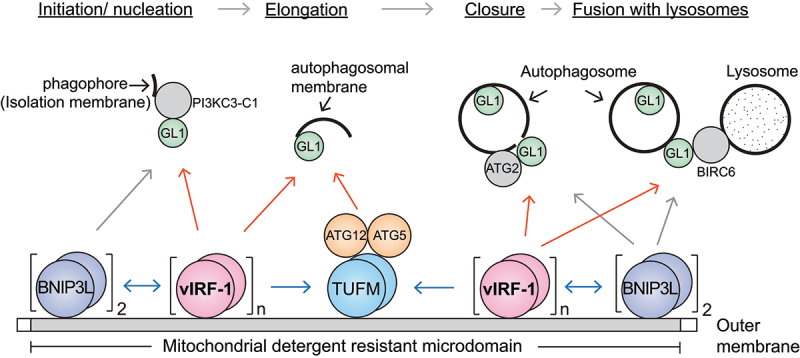
Proposed model of vIRF-1-mediated mitophagy. vIRF-1 is expressed following lytic reactivation and localized to mitochondria by targeting detergent-resistant membrane domains where it interacts with the autophagy proteins BNIP3L, TUFM, and GABARAPL1 (GL1). BNIP3L and vIRF-1 exert on each other for multimerization to be autophagy-competent and may recruit the autophagy machinery to the mitochondria by interacting with GL1. GL1 is known to interact with PI3KC3-C1 (phosphatidylinositol 3-kinase catalytic subunit type 3 complex 1), ATG2, and BIRC6 (baculoviral IAP repeat containing 6). In addition, vIRF-1 may promote autophagosome formation by recruiting ATG12-ATG5 proteins to the mitochondria by promoting the formation of autophagy-competent TUFM (the dimerized form of pre-TUFM).
